# Palmitoylation of the Na/Ca exchanger cytoplasmic loop controls its inactivation and internalization during stress signaling

**DOI:** 10.1096/fj.15-276493

**Published:** 2015-07-14

**Authors:** Louise Reilly, Jacqueline Howie, Krzysztof Wypijewski, Michael L. J. Ashford, Donald W. Hilgemann, William Fuller

**Affiliations:** *Division of Cardiovascular and Diabetes Medicine, Medical Research Institute, College of Medicine, Dentistry, and Nursing, University of Dundee, Dundee, United Kingdom; and ^†^Department of Physiology, University of Texas Southwestern Medical Center, Dallas, Texas, USA

**Keywords:** acylation, patch clamp, resin-assisted capture, ion transport, heart

## Abstract

The electrogenic Na/Ca exchanger (NCX) mediates bidirectional Ca movements that are highly sensitive to changes of Na gradients in many cells. NCX1 is implicated in the pathogenesis of heart failure and a number of cardiac arrhythmias. We measured NCX1 palmitoylation using resin-assisted capture, the subcellular location of yellow fluorescent protein–NCX1 fusion proteins, and NCX1 currents using whole-cell voltage clamping. Rat NCX1 is substantially palmitoylated in all tissues examined. Cysteine 739 in the NCX1 large intracellular loop is necessary and sufficient for NCX1 palmitoylation. Palmitoylation of NCX1 occurs in the Golgi and anchors the NCX1 large regulatory intracellular loop to membranes. Surprisingly, palmitoylation does not influence trafficking or localization of NCX1 to surface membranes, nor does it strongly affect the normal forward or reverse transport modes of NCX1. However, exchangers that cannot be palmitoylated do not inactivate normally (leading to substantial activity in conditions when wild-type exchangers are inactive) and do not promote cargo-dependent endocytosis that internalizes 50% of the cell surface following strong G-protein activation or large Ca transients. The palmitoylated cysteine in NCX1 is found in all vertebrate and some invertebrate NCX homologs. Thus, NCX palmitoylation ubiquitously modulates Ca homeostasis and membrane domain function in cells that express NCX proteins.—Reilly, L., Howie, J., Wypijewski, K., Ashford, M. L. J., Hilgemann, D. W., Fuller, W. Palmitoylation of the Na/Ca exchanger cytoplasmic loop controls its inactivation and internalization during stress signaling.

Cardiac diastole relies on effective removal of Ca from the cytoplasm of ventricular myocytes to allow relaxation and refilling of the ventricles. Removal of Ca occurs as a balanced competition between sarcoplasmic reticulum Ca ATPases (SERCA 2a) and Na/Ca exchangers; Ca pumps sequester Ca into the sarcoplasmic reticulum, and Na/Ca exchangers (NCXs) extrude Ca to the extracellular space (1). In large mammals, SERCA mediates ∼70% and NCX ∼30% of Ca removal (2). NCX can operate in either forward (Ca extrusion) or reverse (Ca influx) modes, depending on membrane potential, the prevailing Na gradient, and the subsarcolemmal free Ca concentration. NCX1 splice variants are widely expressed in both excitable and nonexcitable tissues, and the NCX1.1 splice variant is the major Na/Ca exchanger in cardiac muscle (3). NCX2 and NCX3 are expressed in brain and skeletal muscle; like NCX1, multiple splice variants of NCX3 have been identified (4).

Contractile abnormalities that occur in left ventricular hypertrophy and failure can be related to an imbalance between SERCA and NCX. Most commonly, depletion of intracellular Ca stores occurs because either SERCA activity is decreased or NCX activity is increased (5, 6). Currents generated by NCX, especially when its expression is promoted by cardiac pathologies, can cause arrhythmogenic delayed after depolarizations (7, 8). In a setting of ischemia, or reoxygenation of ischemic tissue, reverse-mode NCX can generate Ca overload, leading to necrotic myocyte death (9). Abnormal function of NCX is described in hypertension (10), cerebral ischemia (11), muscular dystrophy (12), and diabetes (13). Accordingly, the potential of NCX as a therapeutic target is relevant to numerous pathologies besides cardiac pathologies (14–19).

The NCX1 transmembrane architecture consists of 2 inverted repeats, each comprising 5 transmembrane helices (20, 21). NCX1 is regulated by both ions that it transports: a large intracellular loop between TM5 and TM6 facilitates inactivation by Na (22) and mediates secondary activation by Ca *via* 2 Ca binding domains (CBDs) (23, 24). Both inactivation by Na [which does not involve an interaction with the CBDs (25, 26)] and activation *via* occupation of CBDs are strongly modulated by binding of the phospholipid PIP_2_ to cationic sites of the cytoplasmic loop, dubbed the exchanger inhibitory peptide (XIP) domain: PIP_2_ activates NCX mostly by antagonizing Na-dependent inactivation (27, 28). The functional roles of phosphorylation of NCX1 remain controversial (29–31), and no dynamic posttranslational modifications are definitively established to directly regulate NCX1. NCX regulatory mechanisms are discussed in detail elsewhere (32).

The reversible acylation of proteins *via* a thioester bond between a cysteine sulfhydryl side chain and the fatty acid palmitate (palmitoylation) commonly regulates membrane association, subcellular location, trafficking, turnover rate, and enzymatic activity of both peripheral and integral membrane proteins (33). The activities of many ion channels and transporters are regulated by palmitoylation (34). Here, we report that NCX1 is palmitoylated in ventricular muscle at a single cysteine in its large intracellular loop. Palmitoylation does not influence constitutive NCX1 trafficking, but it is required for the complete inactivation of NCX1 following chelation of cytoplasmic Ca and/or anionic phospholipids that activate NCX1. In addition, palmitoylation promotes the participation of NCX1 in membrane domain (*i.e.,* lipid raft)-dependent endocytosis that can be activated by G proteins and large Ca transients.

## MATERIALS AND METHODS

All experiments involving animals were approved by the University of Dundee Welfare and Ethical Use of Animals Committee.

### Materials and antibodies

Anti-NCX1 was from Swant, anti-caveolin 3 and flotillin 2 were from BD Biosciences (San Jose, CA, USA), and anti–green fluorescent protein was from Abcam (Cambridge, MA, USA). The monoclonal antibody α6F raised against the sodium pump α1 subunit by Douglas M. Fambrough (Johns Hopkins University, Baltimore, MD, USA) was obtained from the Developmental Studies Hybridoma Bank developed under the auspices of the *Eunice Kennedy Shriver* National Institute of Child Health and Human Development (NICHD) and maintained by The University of Iowa, Department of Biology (Iowa City, IA, USA).

### Purification of palmitoylated proteins

Palmitoylated proteins were purified using thiopropyl Sepharose in the presence of neutral hydroxylamine after alkylation of free thiols with methyl methanethiosulfonate, as described previously (35). Unfractionated (after methyl methanethiosulfonate block), unbound (not captured by thiopropyl Sepharose in the presence of hydroxylamine), and acylated (captured by thiopropyl Sepharose beads) fractions were routinely analyzed to assess depletion of proteins from the unbound fraction and their enrichment in the acylated fraction.

### Purification of cell surface proteins

Representative fractions of cell surface proteins were prepared by briefly treating cells with 1 mg/ml sulfo-NHS-SS-biotin (Pierce, Rockford, IL, USA) for 10 min at 37°C to biotinylate integral surface membrane proteins with extracellular primary amines, which were subsequently purified using streptavidin Sepharose (GE Healthcare, Waukesha, WI, USA).

### Plasmids, cell lines, and transfection

Canine NCX1.1 cDNA was kindly provided by Professor Godfrey Smith (University of Glasgow, Glasgow, United Kingdom). DsRed-ER was from Clontech (Mountain View, CA, USA) and Grasp65-mCherry was kindly provided by Dr. Jon Lane (University of Bristol, Bristol, United Kingdom). Yellow fluorescent protein (YFP) NCX1 large intracellular loop (YFP-NCX1-IC) was created by inserting canine NCX1.1 cDNA (codons 219 to 761) at the 3′ end of YFP in vector pEFYP-C1 (Clontech). All transfections of plasmid DNA used Lipofectamine 2000 (Invitrogen, Carlsbad, CA, USA) according to the manufacturer’s instructions. Human embryonic kidney (HEK)-derived FT-293 cells expressing tet-inducible wild-type (WT) and C739A NCX1 were generated using the Invitrogen Flip-In T-Rex system.

### Myocytes

Ca-tolerant adult ventricular myocytes were isolated from the hearts of male Wistar rats (250–300 g) by retrograde perfusion of collagenase in the Langendorff mode. Human-induced pluripotent stem cells were differentiated into cardiac myocytes at the University of Dundee iPSC facility using established protocols.

### Microscopy

Images were acquired from cells fixed in 4% paraformaldehyde 24 h after transfection using a Leica SP5 confocal microscope. Acquisition parameters were (excitation/emission, in nanometers): YFP, 519/542; DsRed, 575/644; mCherry, 580/700.

### Electrophysiology

FT-293 cells were removed from dishes by treatment with trypsin (0.1%) and were patch clamped (whole-cell configuration) at 37°C with pipette perfusion and fast bath solution exchange and capacitance measurements as described previously (36). Holding potential was 0 mV, and solutions employed were designed to minimize all membrane currents not carried by NCX. Solution compositions are given in **Table 1**.

**TABLE 1.** Compositions of solutions in electrophysiology experiments

Ingredient	Fig. 4*A*		Fig. 4*B*		Fig. 5*A*		Figs. 5*B* and 7		Fig. 6	
	Pipette	Bath	Pipette	Bath	Pipette	Bath	Pipette	Bath	Pipette	Bath
NaOH (mM)	40	70	0	120 or 0	40	70	40	0	40	120
LiOH (mM)	0	0	0	0 or 120	0	0	0	0	0	0
NMDG (mM)	85	70	120	0	85	70	85	120	85	0
TEA-OH (mM)	15	10	15	10	15	10	15	20	15	15
HEPES (mM)	15	10	15	10	15	10	15	10	15	10
EGTA (mM)	20	0.5	20	0.5	20	0.5	0.5 or 10	0.5	10	0.5
CaCO_3_ (mM)	2 or 10	0	10 or 18	0	0	0	0	0	0	
CaCl_2_ (mM)	0	0 or 4	0	0	0	0 or 4	0.25 or 1	0 or 4	1	0 or 4
MgCl_2_ (mM)	0.5	4 or 0	0.25	4	0.25	4 or 0	0.5	4 or 0	0.5	4
MgATP (mM)	8	0	8	0	8	0	0	0	0	0
TrisATP (mM)	2	0	2	0	2	0	0	0	0	0
Asp to pH (mM)	7.0	7.4	7.0	7.4	7.0	7.4	7.0	7.4	7.0	7.4
AlCl_3_ (mM)	0	0	0	0	0	0	0	0	0.15	0
Free pipette Ca (µM)	0.1 or 0.5		0.5 or 3.0		0		0.5 or 0.1		0.1	

### Sucrose gradient fractionation

Caveolin-enriched buoyant membranes were prepared from ventricular myocytes using a discontinuous sucrose gradient as described previously (37). Following overnight centrifugation the uppermost three 1 ml fractions were discarded, and the next 9 fractions (numbers 4 to 12) collected. Caveolin 3-enriched caveolar membranes were concentrated in fractions 4 and 5.

### Site-directed mutagenesis

Mutagenesis reactions were performed with the Quikchange Lightning and Quikchange Multi Site-Directed Mutagenesis kits (Agilent, Santa Clara, CA, USA). Individual cysteines were mutated to alanines using the Quikchange Lightning Site-Directed Mutagenesis Kit (Agilent). The mutagenesis primer pairs used were as follows: C383A: forward, CAAGGGACCTATCAGGCTCTGGAGAACTGTGGG, reverse, CCCACAGTTCTCCAGAGCCTGATAGCTCCCTTG; C387A: forward, CAGTGTCTGGAGAACGCTGGGACTGTAGCCC, reverse, GGGCTACAGTCCCAGCGTTCTCCAGACACTG; C383A/C387A: forward, GGGACCTATCAGGCTCTGGAGAACGCTGGGACTGTAGCC, reverse, GGCTACAGTCCCAGCGTTCTCCAGAGCCTGATAGGTCCC; C485A: forward, GTTTCTGCGCTCGCTGCCCTGGGATCTCCC, reverse, GGGAGATCCCAGGGCAGCGAGCGCAGAAAC; C557A: forward, GGACTTTGAGGACACTGCTGGAGAGCTCGAATTCC, reverse, GGAATTCGAGCTCTCCAGCAGTGTCCTCAAAGTCC; C731A: forward, GATGATGACGACGATGAAGCTGGAGAGGAGAAGCTG, reverse, CAGCTTCTCCTCTCCAGCTTCATCGTCGTCATCATC; C739A: forward, GGAGAAGCTGCCCTCCGCTTTCGATTATGTGATGC, reverse, GCATCACATAATCGAAAGCGGAGGGCAGCTTCTCC; C914A: forward, CAAGCTCCTCACATCCGCCCTCTTCGTGCTCCTATG, reverse, CATAGGAGCACGAAGAGGGCGGATGTGAGGAGCTTGG.

A cys-less NCX1 intracellular loop (cysteines 383, 387, 485, 557, 731, 739 all mutated to alanine) was created using the Quikchange Multi Site-Directed Mutagenesis Kit, which requires forward primers only. The same mutagenesis reaction also yielded all other mutants described.

## RESULTS

### NCX1 is palmitoylated in cardiac muscle, brain, and kidney

**Figure 1.**
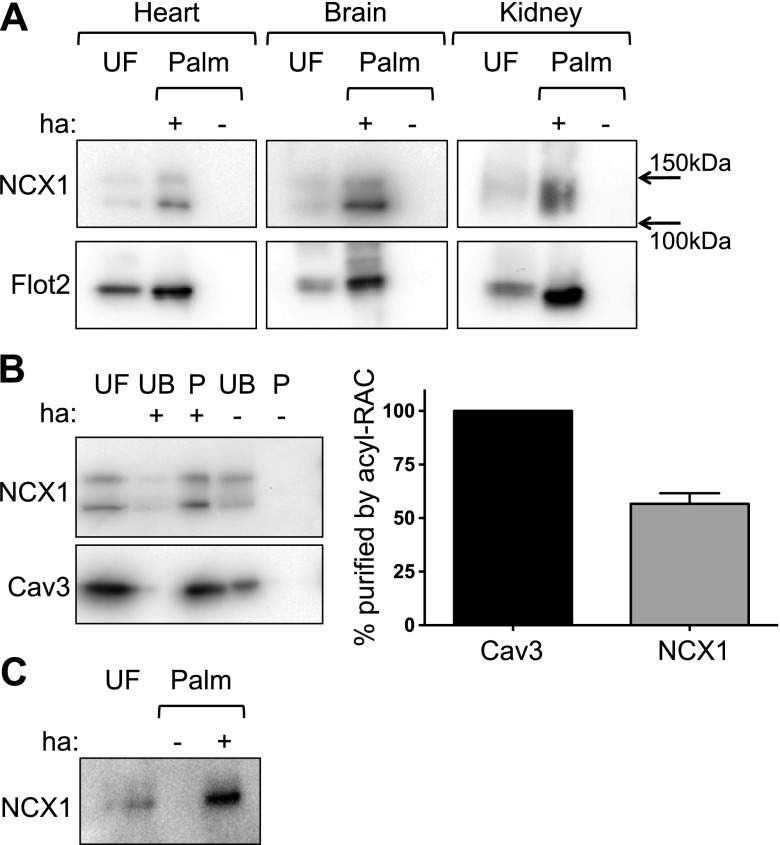
NCX1 is palmitoylated in heart, brain and kidney. *A*) Palmitoylated proteins were purified from homogenates of heart, brain, and kidney and immunoblotted as shown. Note the extended incubation at 40°C during acyl-RAC causes partial aggregation of NCX1 resulting in 2 species of ∼120 and 150 kDa resolved by SDS-PAGE. *B*) Fraction of NCX1.1 palmitoylated in ventricular muscle. A small fraction of NCX1.1 is not palmitoylated in ventricular muscle. NCX1.1 enrichment in acyl-RAC reactions was normalized to the constitutively palmitoylated protein caveolin 3 (Cav3). *n* = 5, mean ± sem shown. *C*) NCX1.1 is palmitoylated in human-induced pluripotent stem cells differentiated into cardiac myocytes. P, proteins purified by acyl-RAC; Palm, proteins purified using acyl-RAC in either the presence (+) or absence (−) of hydroxylamine (ha); UF, unfractionated cell lysate; UB, material not captured by acyl-RAC.

Analysis of the rat neural palmitoyl proteome has previously identified NCX1 and NCX2 as being palmitoylated (38), but did not address whether a biologically meaningful fraction of NCX was modified. We purified palmitoylated proteins using resin-assisted capture of acylated proteins (acyl-RAC) from homogenates of rat brain, kidney, and heart (**Fig. 1*A***). NCX1 was robustly palmitoylated in all tissues examined, implying that multiple splice variants are palmitoylated. We determined what fraction of the cardiac splice variant NCX1.1 is palmitoylated in isolated rat ventricular myocytes by comparing the enrichment of NCX1 following acyl-RAC with the enrichment of the stoichiometrically palmitoylated protein caveolin 3 (39). A small fraction of NCX1 was not purified by acyl-RAC, but all caveolin 3 was captured. The results indicate that ∼60% of NCX1 is palmitoylated in ventricular muscle (Fig. 1*B*). We also confirmed palmitoylation of NCX1.1 in human-induced pluripotent stem cells differentiated into cardiac myocytes (Fig. 1*C*).

### Identification of the NCX1 palmitoylation site

**Figure 2.**
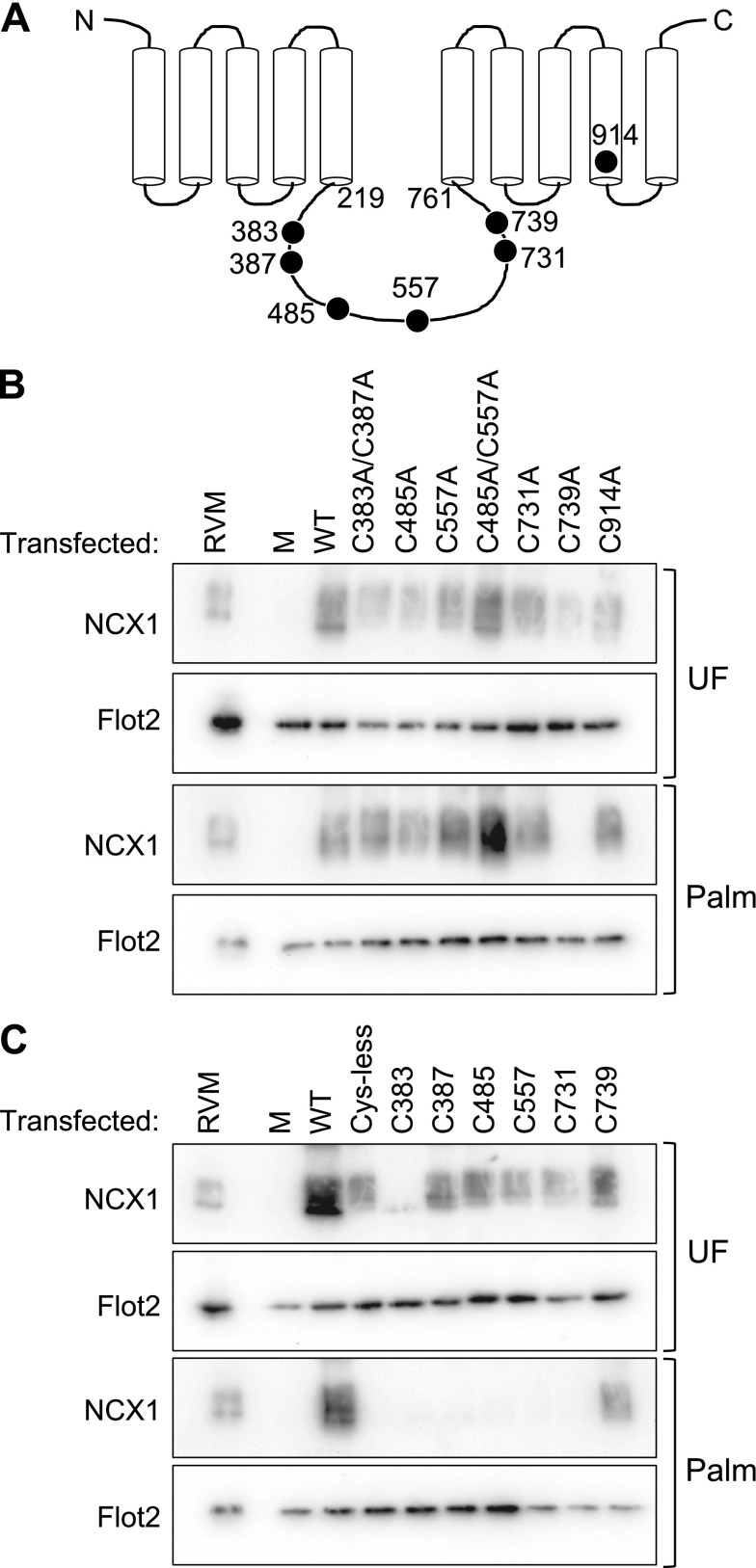
Identification of the NCX1 palmitoylation site. *A*) Membrane topology of mature NCX1.1 following cleavage of the signal peptide. The start and end of the large intracellular loop, and positions of the mutated cysteines are numbered. *B*) Individual cysteines and pairs of cysteines were mutated to alanine and transiently expressed in HEK cells. All mutants were palmitoylated with the exception of C739A. All acyl-RAC reactions were blotted for flotillin 2 (Flot2) to confirm efficient purification of palmitoylated proteins. *C*) Individual cysteines were reintroduced to NCX1 with a cys-less intracellular loop (cysteines 383, 387, 485, 557, 731, and 739 all mutated to alanine). Palmitoylation of NCX1 requires the presence of C739 only. M, mock transfected; Palm, proteins purified using acyl-RAC; RVM, rat ventricular myocytes; UF, unfractionated cell lysate.

To identify the site of palmitoylation in NCX1, individual cysteines predicted to reside in or close to the cytoplasmic domains (see currently accepted topology, **Fig. 2*A***) were mutated to alanine in the NCX1.1 canine cDNA, then expressed in HEK (Fig. 2*B*) and HeLa (not shown) cells. All cysteine-to-alanine mutants investigated displayed similar palmitoylation to WT NCX1 with the exception of mutant C739A, in which palmitoylation was abolished, strongly suggesting that this is the sole site of palmitoylation in NCX1.

To confirm that the presence of C739 is necessary and sufficient for palmitoylation of NCX1 we engineered a cys-less NCX1 in which all cysteines in the large intracellular loop were mutated to alanine. Individual cysteines were added back to the intracellular loop, and palmitoylation was assessed by acyl-RAC following expression in HEK (Fig. 2*C*) and HeLa (not shown) cells. NCX1 was only palmitoylated when C739 was present.

### Impact of palmitoylation on NCX1 trafficking and subcellular localization

**Figure 3.**
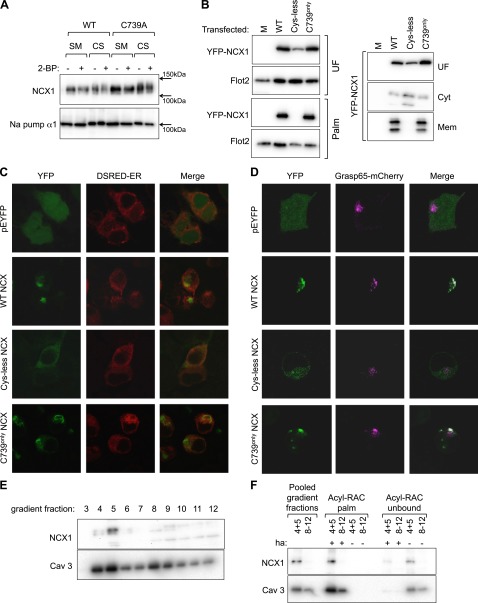
Impact of palmitoylation on NCX1 trafficking and subcellular localization. *A*) Neither pharmacological nor genetic inhibition of NCX1 palmitoylation alters cell surface expression of NCX1 in stably transfected FT-293 cells (2-BP, 100 µM, applied overnight). *B*) Fractionation based on detergent solubility indicates YFP-NCX1-IC is membrane-anchored when palmitoylated in transiently transfected HEK cells. *C*) Subcellular distribution of YFP-NCX1-IC in transiently transfected HEK cells. Cys-less YFP-NCX1-IC is distributed similar to YFP alone. WT and C739^only^ YFP-NCX1-IC localize to an intracellular compartment but do not colocalize with the endoplasmic reticulum marker DsRed-ER. *D*) WT and C739^only^ YFP-NCX1-IC colocalize with the Golgi marker Grasp65-mCherry in transiently transfected HEK cells. *E*) NCX1 localizes to buoyant caveolin enriched microdomains in rat ventricular myocytes. *F*) Both palmitoylated and nonpalmitoylated NCX1 are present in buoyant caveolin enriched microdomains prepared from rat ventricular myocytes. 2-BP, 2-bromopalmitate; CS, cell surface protein; SM, starting cell lysate.

The pharmacological specificity of 2-bromopalmitate, widely used as an inhibitor of Asp-His-His-Cys motifs containing palmitoyl acyl transferases, has recently been questioned (40). We therefore used both genetic and pharmacological approaches to assess the impact of palmitoylation on trafficking of NCX1 through the secretory pathway in FT-293 cells stably expressing WT and C739A NCX1. Neither 2-bromopalmitate (100 µM, applied overnight) nor mutation C739A influenced cell surface localization of NCX1 (assessed using membrane impermeable amine-reactive biotinylation reagents, **Fig. 3*A***).

We next investigated the subcellular compartment in which NCX1 is palmitoylated and the consequences of palmitoylation by using a YFP fusion protein of the YFP-NCX1-IC. WT, cys-less, and C739^only^ loops fused to the C terminus of YFP were expressed by transient transfection in HEK cells. We used a simple fractionation protocol based on detergent solubility to prepare cytosolic (digitonin soluble), membrane (Triton X-100 soluble), and insoluble fractions. Cys-less YFP-NCX1-IC was entirely cytosolic (Fig. 3*B*), whereas WT and C739^only^ loops were found in both cytosolic and membrane fractions, indicating that the presence of the palmitoylation site at C739 was sufficient to provide a membrane anchor in the absence of NCX1 transmembrane domains. The subcellular location of all 3 loops and their colocalization with fluorescent endoplasmic reticulum (DsRed-ER, Fig. 3*C*) and Golgi (Grasp65-mCherry, Fig. 3*D*) marker proteins were also investigated. The subcellular distribution of cys-less YFP-NCX1-IC essentially matched YFP alone, whereas WT and C739^only^ loops were localized to punctate intracellular structures that colocalized with a Golgi but not an endoplasmic reticulum marker.

Palmitoylation is proposed to partition proteins to detergent-resistant membranes such as caveolae (41), so we investigated localization of NCX1 to cardiac caveolae. NCX1 is reported to localize to caveolae by some but not all researchers (42, 43). We found that NCX1 robustly localizes to buoyant caveolin-enriched microdomains prepared from rat ventricular myocytes using a standard discontinuous sucrose gradient (Fig. 3*E*). Buoyant (pooled fractions 4 and 5) and dense (pooled fractions 8–12) membranes were used in acyl-RAC experiments to separate palmitoylated from nonpalmitoylated proteins (Fig. 3*F*). Although the majority of NCX1 localizes to buoyant membranes in rat ventricular myocytes, we found both palmitoylated and nonpalmitoylated NCX1 in these membranes, suggesting that palmitoylation is not required for localization of NCX1 to cardiac caveolae.

### Functional effect of palmitoylation on NCX1

**Figure 4.**
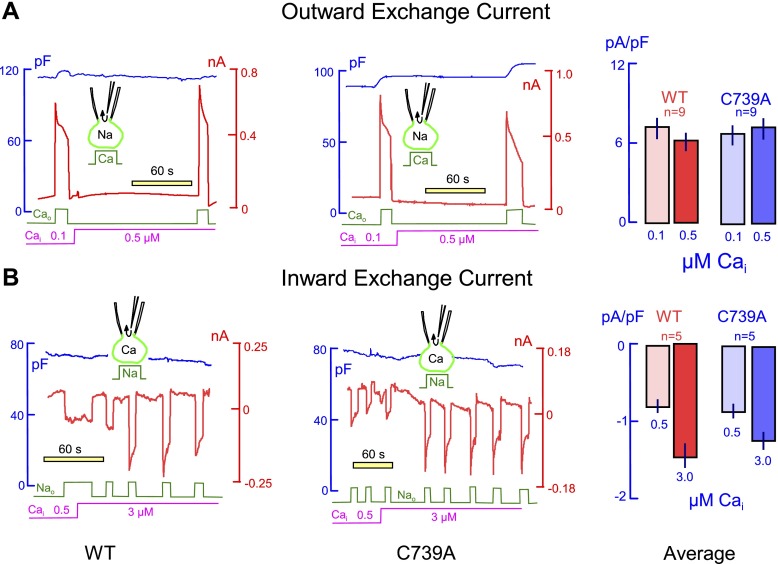
Effect of palmitoylation on NCX1 function. Stably transfected FT-293 cells expressing WT and C739A NCX1 were voltage clamped in the whole-cell mode. Current traces are shown in red, capacitance traces in blue, changes in extracellular solution indicated in green, and changes in pipette solution in pink. *A*) Outward NCX1 currents in the presence of both 0.1 and 0.5 µM intracellular Ca were activated by stepping extracellular Ca from 0 to 4 mM in cells expressing WT (left) and C739A exchangers (center). No differences in outward exchanger current were observed in WT or nonpalmitoylatable NCX1 (right). *B*) Inward NCX1 currents in the presence of both 0.5 and 3 µM intracellular Ca were activated by the application of 120 mM extracellular Na in exchange for Li in cells expressing WT (left) and C739A exchangers (center). No differences in inward exchanger currents were observed in WT or nonpalmitoylatable NCX1 (right). Current transients that occur during the activation of inward currents by extracellular Na likely reflect cytoplasmic Ca depletion, namely because the buffer capacity of EGTA is weak when it is nearly saturated with Ca to generate 3 µM free Ca.

We used whole cell voltage clamp of FT 293 cells stably expressing WT and C739A NCX1 to determine the impact of palmitoylation on NCX1 function (**Fig. 4**). Using cytoplasmic solutions that were heavily Ca-buffered, outward NCX1 currents (activated by the application of 4 mM extracellular Ca) were measured in the presence of both 0.1 and 0.5 µM free intracellular Ca (Fig. 4*A*). Inward NCX1 currents (activated by the application of 120 mM extracellular Na in exchange for Li) were measured in the presence of 0.5 and 3 µM free intracellular Ca (Fig. 4*B*). No quantitative or qualitative differences in either outward or inward exchanger currents were identified between cells expressing WT and C739A NCX1.

### Palmitoylation and NCX1 inactivation

**Figure 5.**
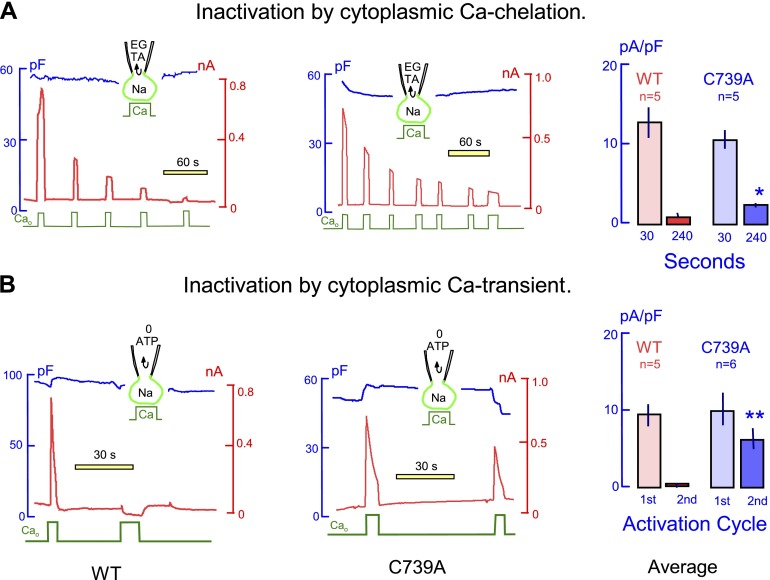
Effect of palmitoylation on NCX1 inactivation by Ca depletion and excess. Stably transfected FT-293 cells expressing WT and C739A NCX1 were voltage clamped in the whole-cell mode. Current traces are shown in red, capacitance traces in blue, and changes in extracellular Ca indicated in green. Outward NCX1 currents were activated by stepping extracellular Ca from 0 to 4 mM in cells expressing WT (left) and C739A exchangers (center). *A*) Effect of removal of intracellular Ca with EGTA. NCX1 inactivation is significantly slower in C739A NCX1 compared with WT: 240 s after application of EGTA a significantly greater current remains in cells expressing C739A NCX1 (right). *P < 0.05 *vs.* WT. *B*) Effect of PIP_2_ depletion by a large Ca transient in the absence of intracellular ATP. In this protocol, the magnitude of the initial NCX1 current remains stable for periods of many minutes although the cytoplasmic ATP concentration is nominally zero. The initial activation cycle results in complete NCX1 inactivation in WT but not C739A-expressing cells (right). **P < 0.01 *vs.* WT.

**Figure 6.**
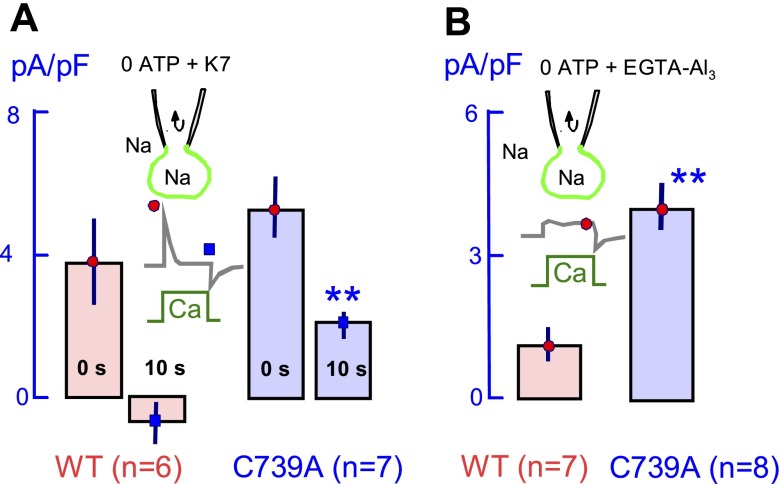
Effect of palmitoylation on NCX1 inactivation by masking anionic phospholipid head groups. Stably transfected FT-293 cells expressing WT and C739A NCX1 were voltage clamped in the whole-cell mode using ATP-free, highly Ca-buffered cytoplasmic solutions (10 mM EGTA with 1 mM Ca). *A*) Using a cytoplasmic solution containing 20 µM heptalysine (K7) to bind head groups of anionic phospholipids, outward exchange currents were activated by stepping extracellular Ca from 0 to 4 mM in cells expressing WT (left) and C739A exchangers (right). Current magnitudes are given for peak currents and current remaining after 10 s. Residual currents are highly significantly increased in cells expressing C739A compared with WT exchangers. *B*) Using a cytoplasmic solution containing 0.15 mM Al^3+^ (in the presence of 10 mM EGTA) to bind PIP_2_ head groups, outward exchange currents were strongly suppressed in cells expressing WT exchangers (left) but remained robust in cells expressing C739A exchangers (right). **P < 0.01.

NCXs contribute a substantial proportion of Na load in cardiac muscle, and Na-dependent inactivation of NCXs may decrease myocyte Na load during metabolic stress as part of a cell program to shift Ca turnover to internal recycling and away from turnover across the sarcolemma (44). We measured the impact of palmitoylation on the transition of NCX1 to inactivated states by inducing inactivation in 4 different ways (**Figs. 5** and **6**). First, complete chelation of cytoplasmic Ca completely inactivates NCX1, probably because Ca binding to CBDs in the NCX1 large intracellular loop antagonizes Na-dependent inactivation (45). Using 20 mM EGTA without added Ca on the cytoplasmic side, exchange currents declined over the course of 4 min to negligible values in cells expressing WT exchangers but to 22% of initial values in cells expressing C739A exchangers (Fig. 5*A*, *P* < 0.05). Second, in the absence of intracellular ATP, a large Ca transient inactivates NCX1 by causing depletion of PIP_2_, through the activity of PLCs (46). In this case, a single large Ca transient (induced by activating reverse exchange current in the presence of a low intracellular EGTA concentration), results in >90% inactivation of NCX1 current at subsequent activation episodes (Fig. 5*B*). This nearly complete inactivation was reduced to <30% in cells expressing C739A NCX1 (*P* < 0.01). We therefore conclude that palmitoylation facilitates and may be required for complete inactivation of NCX1 by both removal of cytoplasmic Ca and by loss of PIP_2_.

As a means to cause inactivation of NCX1 without changing cytoplasmic free Ca, we included polylysine in ATP-free cytoplasmic solutions to bind anionic phospholipids prior to activation of exchange current by Ca (47). Figure 6*A* shows results with 20 μM heptalysine added to cytoplasmic solutions. The initial exchange current activated by extracellular Ca in cells expressing WT exchangers is decreased by more than 50% from those in Fig. 5, and current decays within seconds to negligible values during application of extracellular Ca. The average currents in cells expressing C739A-mutant exchangers decline substantially less the WT currents (*P* < 0.01) to about 30% of peak currents.

Finally, we exploited that fact that EGTA/aluminum chelates effectively provide Al^3+^ to bind to PIP_2_ head groups with extraordinarily high affinity, thereby masking PIP_2_ head groups in a specific manner (27). Using ATP-free pipette solutions with 10 mM EGTA and 0.15 mM Al^3+^, the steady-state exchange currents in WT-expressing cells are reduced to 0.81 ± 0.5 pAmp/pFarad (∼10% of routine values), and currents obtained in C739A-expressing cells are 4-fold greater (3.7 pAmp/pFarad, *P* < 0.01; nearly 50% of routine values). Therewith, results from 4 different experimental protocols concur that inactivation of NCX1 is decreased by the C739A mutation.

### NCX1 palmitoylation and membrane endocytosis

**Figure 7.**
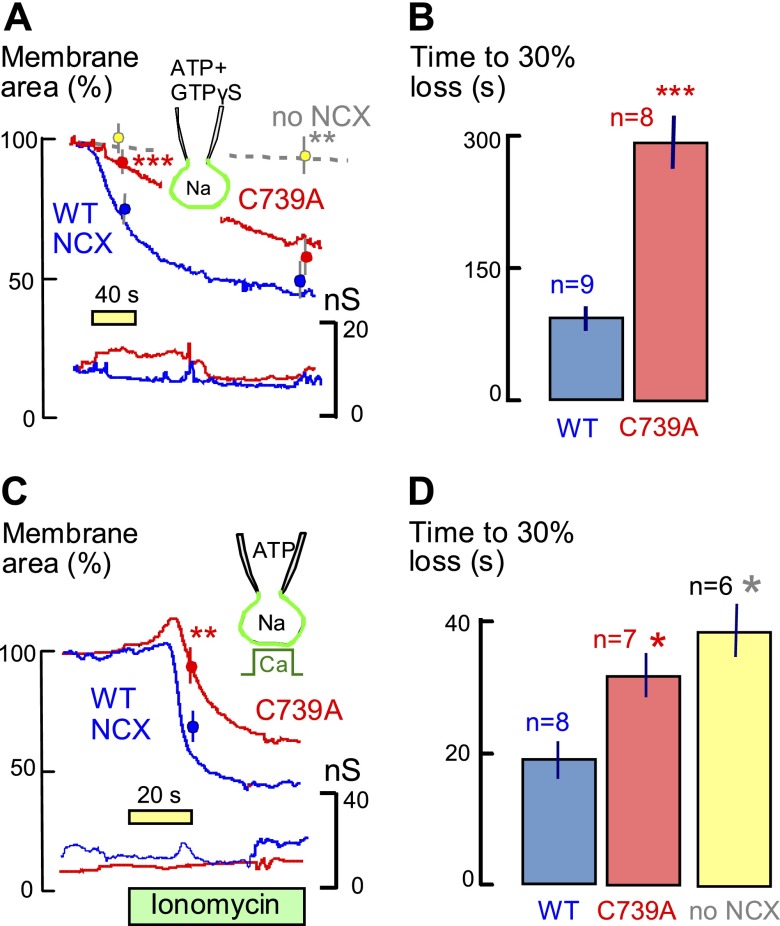
Impact of palmitoylation of NCX1 on massive endocytosis occurring in response to excessive G-protein activation and Ca influx. Stably transfected FT-293 cells expressing WT and C739A NCX1 were voltage clamped in the whole-cell mode with 8 mM MgATP in the pipette solution. *A*) Using a highly Ca-buffered cytoplasmic solution (10 mM EGTA with 1 mM Ca) with 0.25 mM GTPγS, cells expressing WT exchangers internalize 33 ± 3% of their membrane within 40 s of establishing the whole cell configuration, whereas cells expressing C739A exchangers internalize only 10 ± 2%. (*P* < 0.001). At 3 min, the difference between exchanger-expression cells is not significant, and membrane loss in cells without exchangers is negligible. *B*) Quantitation of endocytosis as the time required to decrease membrane area by 30%. C739A-expressing cells internalize at a rate that is >3 times less than cells expressing WT exchangers. *C*) Using a weakly Ca-buffered cytoplasmic solution (0.5 mM EGTA with 0.25 mM Ca), extracellular Ca (2 mM) is applied with 7 μM ionomycin to promote Ca influx. In WT-expressing cells, small initial exocytic responses (*i.e.,* with increasing membrane area) are followed by loss of 50% of the cell surface within about 20 s. In C739 cells, the exocytic responses are larger and endocytic responses are delayed. The loss of membrane area in cells expressing WT exchangers amounts to 33 ± 4%, and loss of membrane in C739A-expressing cells amounts to only 13 ± 6% (*P* < 0.01). *D*) Quantitation of Ca-induced endocytic responses as the time required to remove 30% of the cell surface. Endocytosis times are increased by >50% in C739A cells, compared with WT cells (*P* < 0.05), and endocytosis times are doubled in cells that do not express NCX1 (*P* < 0.01). *P < 0.05, **P < 0.01, ***P < 0.001.

Given that palmitoylation does not affect exchanger ocalization in HEK cells, we tested whether NCX1 palmitoylation might affect endocytosis by adapter-independent mechanisms that can occur during cell stress in a palmitoylation-dependent manner (48). Specifically, it has been demonstrated for Na/K pumps that palmitoylation of the accessory subunit phospholemman can promote adapter-independent endocytosis that is enhanced by overexpression of phospholemman and therefore is cargo-dependent (49). In **Fig. 7**, we employed 2 protocols that result in endocytosis of about 50% of the cell surface, and in both protocols the C739A mutation strongly decreases endocytosis monitored as a loss of membrane capacitance. In Fig. 7*A* we describe that the nonhydrolyzable GTP analog, GTPγS, activates massive endocytosis in WT-expressing cells with free cytoplasmic Ca clamped to 0.1 µM. Clearly, G-protein activation can powerfully promote adapter-independent endocytosis (50) when: 1) clathrin is blocked by potassium-free cytoplasmic solutions (36); 2) the GTP-hydrolyzing endocytic protein, dynamin, is inhibited by GTPγS (51); and 3) palmitoylated NCX1 exchangers are highly expressed in the surface membrane. As shown in Fig. 7*A*, fully 50% of cell area is lost within 3 min when WT-expressing cells are opened with the Ca-buffered, GTPγS-containing pipette solution. Although nearly the same amount of membrane is lost in C739 mutant cells over time, the rate of membrane loss is decreased by more than 3-fold (Fig. 7*B*, *P* < 0.01). This form of endocytosis is strongly cargo-dependent, as illustrated by the near absence of endocytosis in FT-293 cells expressing no exchangers (Fig. 7*A*, dotted line). As shown in Fig. 7*C* using a cytoplasmic solution with low Ca buffering power, more than 50% of cell membrane is also lost *via* Ca-dependent endocytosis when NCX-expressing cells are exposed to extracellular ionomycin and Ca. The time required to remove 30% of the cell surface (Fig. 7*D*) was increased from 19 s in WT-expressing cells to 31 s in C739A-expressing cells and to 38 s in FT-293 cells that do not express exchangers. In conclusion, these results demonstrate by 2 protocols that overexpression of NCX1 promotes adapter-independent endocytosis and that NCX1 palmitoylation further promotes this form of endocytosis, presumably by promoting the formation of lipid–protein domains in the cell surface.

## DISCUSSION

In this article, we have documented that NCX1 is substantially palmitoylated in cardiac muscle. Nonpalmitoylatable mutant exchangers do not inactivate fully when cytoplasmic Ca is chelated or when the negative charges of anionic phospholipids are neutralized. In addition, the ability of exchangers to promote adapter-independent, cargo-dependent endocytosis is substantively decreased in a nonpalmitoylatable NCX1 mutant. Notably, this mutant still localizes to the surface membrane and functions in a nominally normal fashion. Thus, palmitoylation tunes NCX function in only a subset of cellular circumstances, specifically circumstance of cell stress in which exchanger function can be pivotal for cell survival or demise. Palmitoylation occurs early in the lifetime of NCX1, so we hypothesize that depalmitoylation provides a mechanism to regulate exchanger function after insertion into the cell surface, as well as to regulate its removal from the cell surface in cell stress.

### Functional effects of palmitoylation

NCX is regulated by both ions it transports *via* reactions that involve its large intracellular loop. Ca-dependent activation is mediated by two CBDs: Ca binding to CBD1 (residues 361–461) with high (<1 µM) affinity activates the exchanger, and Ca binding to CBD2 (residues 492–592) with lower affinity relieves Na-dependent inactivation (47). Na-dependent inactivation requires the XIP region of the intracellular loop (residues 219–238) (52) and is reversed by Mg-ATP–dependent synthesis of PIP_2_ (27, 53). Inhibition by XIP is proposed to involve a direct interaction between XIP and residues 562–679, within CBD2 (54). NCX1 in our engineered cell lines is activated by cytoplasmic Ca with very high Ca affinity, similar to NCX1 in intact cardiac myocytes (55), and in contrast to results for excised membrane patches, in which 1 log unit higher free Ca is required to activate the outward exchange current (56).

We observed no difference in the activities of WT and unpalmitoylatable exchangers when examined with free cytosplasmic Ca buffered to 0.1 µM or greater. We therefore suggest that the functional effect of palmitoylation on NCX1 inactivation is achieved through an influence on the interaction between XIP and the intracellular loop. Unpalmitoylatable NCX1 does not inactivate following PIP_2_ depletion or chelation, so palmitoylation at C739 seems to be required for the XIP domain to fully inhibit exchange activity. One possibility is that palmitoylation enhances the propensity of anionic lipids to bind the XIP domain and thereby prevent it from inactivating exchange activity.

The extent of NCX palmitoylation varies depending on the cell type examined, being most robust in ventricular myocytes and least robust in cultured cells (Supplemental Fig. 1). The extent of Na-dependent inactivation also varies significantly in different experimental models (53, 57). Therefore, the physiologic roles of Na-dependent inactivation and its relief by PIP_2_ have remained enigmatic (44). Our data suggest that these disparities may reflect differences in the extent of NCX1 palmitoylation in the different models, caused by differences in acyl transferase or thioesterase expression or activity in different cell types. Palmitoylation is clearly not required for trafficking of NCX1, so cell surface localized nonpalmitoylated NCX1 has likely been studied in a number of investigations. As well as displaying prominent Na-dependent inactivation, squid NCX1 in giant axon preparations was extensively characterized in a number of classic early studies on the regulation of NCX1 (58). Notably the NCX1 palmitoylation site is conserved in some invertebrates, including squid (discussed below).

Although X-ray and NMR structures of CBD1 and CBD2 in the NCX1 large intracellular loop have been obtained (59), no structural information currently exists about the region of this loop containing the palmitoylation site. Disorder and secondary structure prediction algorithms (not shown) indicate that Cys739 (but not the neighboring Cys731) lies in an ordered region of this loop (60), but that it is less than 25% solvent exposed (61), suggesting that weak membrane association may precede its palmitoylation.

### Palmitoylation regulates multiple NCX1 interactions with the surface membrane

The presence of a membrane anchor at position C739 can be expected to influence the tilt of multiple interacting transmembrane domains in NCX1 (52). Transitions between outward- and inward-facing confirmations of a bacterial NCX homolog are proposed to require movement of the loosely packed helices transmembrane domains 1 and 6 (21). It is rather surprising therefore that the normal function of NCX1 is not more markedly modified by the palmitoylation at C739. Rather, our work suggests that palmitoylation changes primarily the relationship of NCX1 to the lipid bilayer with consequences for NCX1 regulation and participation in membrane domains. Specifically, the full inactivation of NCX upon depletion of cytoplasmic Ca, by nonspecific masking of anionic phospholipids, by depletion of PIP_2_, and by specific masking of PIP_2_ head groups (Figs. 5 and 6) all require NCX palmitoylation. Thus it is likely that unpalmitoylatable NCX1 is active in PIP_2_-free intracellular membrane compartments but WT NCX1 is not. Potentially, the complete inactivation of NCX during its processing and trafficking prevents NCX from depleting Ca gradients in internal membrane compartments where Na gradients are small or minimal (62). Hence, NCX inactivation as a result of its palmitoylation in the Golgi apparatus may preserve this intracellular Ca store (63). That said, we found no influence of unpalmitoylatable NCX1 on cell growth rates or on expression of markers of endoplasmic reticulum stress (Supplemental Fig. 2). This effect of palmitoylation on lipid regulation of an ion transporter bears similarities to the regulation of the sodium pump by its palmitoylated accessory subunit phospholemman, which appears to rely on palmitoylation of phospholemman modulating regulatory lipid interactions with the pump (35, 64). A large number of ion channels and transporters are regulated by PIP_2_, as well as other phospholipids (65), and a similarly large number are also palmitoylated (34). As the only reversible lipid modification, palmitoylation may provide a means to reversibly sensitize channels and transporters to regulation by their phospholipid environment.

In addition to modulating the immediate interactions of NCX1 transporters with phospholipids, our results indicate that palmitoylation of NCX1 promotes its participation in membrane domains that can be internalized in a cargo-dependent manner without involvement of classic endocytic proteins (36). These results confirm that palmitoylation can promote membrane protein/lipid phase separations whereby more ordered domains can be preferentially internalized in many cell types (48). Cells expressing the C739A exchanger mutant have similar exchange activities as those expressing WT NCX1, and cell surface areas are not impressively changed, so it is unlikely that this form of endocytosis is occurring constitutively in the cells employed in this study. The most obvious situation in which this form of endocytosis will play a major role in muscle is the response of myocytes to Ca overload. However, the fact that equally large endocytic responses can occur in the absence of Ca transients, namely when G proteins are highly activated by GTPγS (Fig. 7*A*), suggests that other types of cell stress can also activate these endocytic processes to dramatically remodel the cell surface.

### Palmitoylation of other members of the cation/Ca^2+^ exchanger superfamily

**Figure 8.**
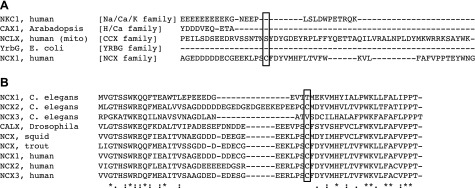
Conservation of the NCX palmitoylation site. *A*) The hydrophilic cytosolic loops of representative members of the cation/Ca2^+^ exchanger superfamily were aligned using Clustal Omega (67). The NCX palmitoylated cysteine (boxed) is not conserved in other branches of this superfamily. *B*) NCX family members from vertebrates and invertebrates were aligned using Clustal Omega. The NCX1 palmitoylated cysteine (boxed) is conserved among all NCX isoforms in all vertebrates, as well as squid and the *Drosophila* exchanger Calx, but it is not present in NCX1 or NCX3 from *C. elegans*. In NCX variants that contain the palmitoylated cysteine, glutamate at −6, proline at −2, tyrosine at +3, and histidine at +6 are 100% conserved.

NCX1 is a member of the cation/Ca exchanger superfamily, which is defined by the presence of highly conserved α-repeat regions within 2 membranous hydrophobic domains, which are separated by a hydrophilic cytosolic loop rich in acidic amino acids. This superfamily of 147 transporters from both eukaryotes and prokaryotes consists of 5 distinct protein families (66). We used Clustal Omega (67) to align the hydrophilic regions of representative members (mammalian, where available) of each family (**Fig. 8*A***); the palmitoylated cysteine in NCX1 is not found in the other mammalian family members (Na/K/Ca exchangers or the mitochondrial exchanger NCLX), or in the H/Ca exchanger or YRBG families. No homologous position exists in the crystallized NCX homolog NCX_Mj, which is short (7 amino acids) and disordered (21).

Alignment of NCX family members (Fig. 8*B*) indicates the NCX1 palmitoylated cysteine (and some other local sequence features) is conserved among all NCX isoforms in all vertebrates, as well as squid and the *Drosophila* exchanger Calx, but it is only present in NCX2 from *Caenorhabditis* *elegans*. We therefore suggest that this palmitoylation site is an adaptation in vertebrates and higher invertebrates to regulate NCX activity.

## CONCLUSIONS

Although palmitoylation of NCX1 in its large intracellular loop occurs in the secretory pathway, it is surprisingly not required for NCX trafficking or function in our HEK overexpression system. Palmitoylation regulates NCX1 inactivation, possibly influencing the ability of the XIP region to inhibit exchanger activity, and palmitoylation promotes the participation of NCX1 in membrane domains that can be internalized in a cargo-dependent fashion.

## Abbreviations

acyl-RAC: resin-assisted capture of acylated proteins
CBD: Ca-binding domain
HEK: human embryonic kidney
NCX: Na/Ca exchanger
YFP: yellow fluorescent protein
YFP-NCX-IC: yellow fluorescent protein fused to the NCX1.1 intracellular loop
WT: wild-type
XIP: exchanger inhibitory peptide
